# Changes in Pediatric Inpatient Capabilities and Emergency Department Pediatric Readiness

**DOI:** 10.1001/jamanetworkopen.2025.8277

**Published:** 2025-05-01

**Authors:** Ashley A. Foster, Jonathan Eisenberg, Rachel Crady, Hilary A. Hewes, Katherine E. Remick

**Affiliations:** 1 Department of Emergency Medicine, University of California, San Francisco; 2 Department of Pediatrics, Dell Medical School, University of Texas at Austin; 3 Department of Pediatrics, University of Utah, Salt Lake City

## Abstract

This cross-sectional study evaluates changes in pediatric inpatient capabilities among hospitals and examines the association with pediatric readiness.

## Introduction

Most children in the US are evaluated in general emergency departments (ED)s that see fewer than 10 to 15 pediatric patients per day, where pediatric-specific resources may vary.^1,2^ Changes in pediatric inpatient capabilities, particularly the loss of pediatric-specific inpatient units, may negatively impact hospital resources extending to the ED.^3^ The 2013 and 2021 National Pediatric Readiness Project (NPRP) Assessments provide a snapshot of ED pediatric readiness to care for critically ill and injured children, measured by the weighted Pediatric Readiness score (wPRS), a validated measure linked to mortality outcomes.^2,4,5^ This study evaluates changes in pediatric inpatient capabilities among hospitals that completed both assessments and examines the association with Pediatric Readiness.

## Methods

This retrospective cross-sectional study uses data from hospitals that completed both 2013 and 2021 NPRP assessments.^2,4^ The primary exposure was net change in pediatric inpatient capabilities, defined by presence or absence of a pediatric ward, nursery, neonatal intensive care unit (NICU), pediatric intensive care unit (PICU), and adult ward or adult intensive care unit (ICU) that accepts pediatric patients. Changes were measured by net difference in these capabilities between assessments. The primary outcome was the wPRS (reported as median [IQR]). Additional methods are described in the eMethods in Supplement 1. This study was approved by the institutional review board at University of Utah with a waiver of informed consent and conducted in accordance with Strengthening the Reporting of Observational Studies in Epidemiology (STROBE) reporting guidelines. Participation in the assessment was voluntary and score are only shared publicly in aggregate. Differences in pediatric inpatient capabilities between 2013 and 2021 were analyzed using Wilcoxon signed-rank tests to assess changes in wPRS. All tests were 2-sided, and *P* < .05 was considered statistically significant. Data were analyzed from August 2023 to November 2024 year using SAS software version 9.4 (SAS Institute).

## Results

Between 2013 and 2021, 1029 of the 2772 participating hospitals (37.1%) experienced a net decline in pediatric inpatient capabilities, while 701 (25.3%) experienced a net gain (Table). Hospitals with decreased capabilities had a median (IQR) wPRS of 68.4 (57.4-82.6), compared with 68.8 (58.3-84.5) for those with no change and 71.5 (60.5-86.0) for those with increased capabilities (χ^2^_2_ = 11.35; *P* = .003). Overall, there was a reduction in hospitals with pediatric wards, pediatric ICUs, and adult wards accepting pediatric patients (Figure).

**Table.** Study Sample Emergency Department (ED) and Hospital Characteristics

Characteristic	Hospitals, No. (%)				*P* value
	Total study sample (n = 2772)	Change in pediatric inpatient capabilities, 2013-2021			
		Decrease (n = 1029)	None (n = 1042)	Increase (n = 701)	
Urbanicity					
Urban	1655 (59.7)	584 (35.3)	591 (35.7)	480 (29.0)	<.001
Suburban	246 (8.9)	103 (41.9)	80 (32.5)	63 (25.6)	
Rural	563 (20.3)	231 (41.0)	229 (40.7)	103 (18.3)	
Remote	308 (11.1)	111 (36.0)	142 (46.1)	55 (17.9)	
Hospital configuration					
General hospital	1680 (60.6)	613 (36.5)	555 (33.0)	512 (30.5)	<.001
Children’s hospital within a general hospital	115 (4.1)	39 (33.9)	40 (34.8)	36 (31.3)	
Children’s hospital	60 (2.2)	20 (33.3)	31 (51.7)	9 (15.0)	
Critical access	811 (29.3)	321 (39.6)	359 (44.3)	131 (16.2)	
Microhospital^a^	22 (0.8)	11 (50.0)	7 (31.8)	4 (18.2)	
Satellite ED	33 (1.2)	12 (36.4)	20 (60.6)	1 (3.0)	
Freestanding ED	11 (0.4)	3 (27.3)	8 (72.7)	0	
Other	40 (1.4)	10 (25.0)	22 (55.0)	8 (20.0)	
Trauma center designation					
No	1412 (50.9)	526 (37.3)	559 (39.6)	327 (23.2)	.02
Yes	1360 (49.1)	503 (37.0)	483 (35.5)	374 (27.5)	
Indian or tribal hospital					
No	2742 (98.9)	1023 (37.3)	1024 (37.3)	695 (25.3)	.03
Yes	30 (1.1)	6 (20.0)	18 (60.0)	6 (20.0)	
Region					
Midwest	930 (33.5)	367 (39.5)	352 (37.8)	211 (22.7)	<.001
Northeast	233 (8.4)	89 (38.2)	92 (39.5)	52 (22.3)	
South	923 (33.3)	401 (43.4)	326 (35.3)	196 (21.2)	
West	634 (22.9)	150 (23.7)	252 (39.7)	232 (36.6)	
Unknown	52 (1.9)	22 (42.3)	20 (38.5)	10 (19.2)	
ED configuration					
General ED	2500 (90.2)	932 (37.3)	939 (37.6)	629 (25.2)	.01
Separate pediatric ED	188 (6.8)	72 (38.3)	57 (30.3)	59 (31.4)	
Pediatric ED	69 (2.5)	20 (29.0)	38 (55.1)	11 (15.9)	
Other	15 (0.5)	5 (33.3)	8 (53.3)	2 (13.3)	
Pediatric patient volume category (patients/y)					
Low (<1800)	1391 (50.2)	517 (37.2)	580 (41.7)	294 (21.1)	<.001
Medium (1800-4999)	852 (30.7)	325 (38.1)	291 (34.2)	236 (27.7)	
Medium-High (5000-9999)	301 (10.9)	110 (36.5)	79 (26.2)	112 (37.2)	
High (≥10 000)	228 (8.2)	77 (33.8)	92 (40.4)	59 (25.9)	
PECC					
No PECC	1474 (53.2)	565 (38.3)	554 (37.6)	355 (24.1)	.14
Nurse PECC	251 (9.1)	97 (38.6)	99 (39.4)	55 (21.9)	
Physician PECC	236 (8.5)	84 (35.6)	78 (33.1)	74 (31.4)	
Nurse and physician PECC	811 (29.3)	283 (34.9)	311 (38.3)	217 (26.8)	
Weighted pediatric readiness score, median (IQR)	69.1 (58.6-84.1)	68.4 (57.4-82.6)	68.8 (58.3-84.5)	71.5 (60.5-86.0)	.003

Abbreviation: PECC, pediatric emergency care coordinator.

^a^ A microhospital is a “small scale inpatient facility that typically maintains 8-15 beds for observation and short-stay use for low-acuity patients” (National Pediatric Readiness Project Pediatric Readiness Assessment, 2021).

**Figure.**
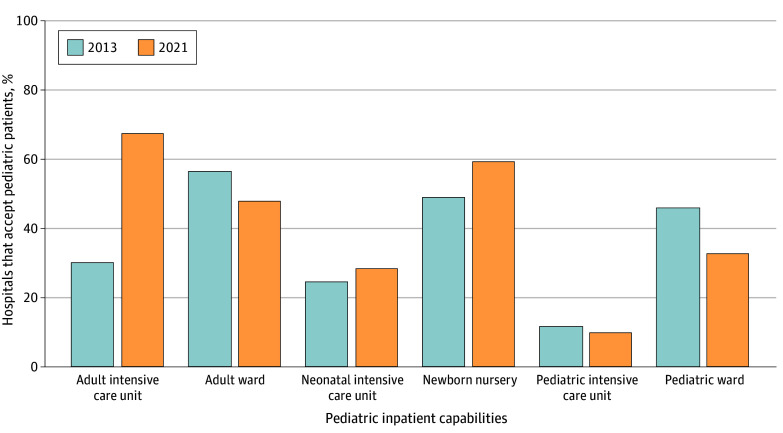
Pediatric Inpatient Capabilities, 2013 and 2021 Bars represent percentage of hospital inpatient units that accept pediatric patients from 2013 and 2021 National Pediatric Readiness Project Assessments. χ^2^ tests of year and pediatric inpatient capability produced *P* < .001.

Hospitals with an addition of pediatric ward (median [IQR], 2013: 64.2 [52.1-78.8]; 2021: 70.8 [59.3-84.6]; *P* = .01), NICU (median [IQR], 2013: 73.2 [58.3-85.9]; 2021: 75.6 [63.7-90.5]; *P* = .01), or PICU (median [IQR], 2013: 86.6 [63.5-93.7]; 2021: 90.6 [77.9-96.1]; *P* = .04) capabilities experienced significant increases in wPRS. Conversely, hospitals with a loss of PICU capabilities had a significant decline in wPRS (median [IQR], 2013: 83.0, [66.7-93.8]; 2021: 71.0, [61.8-86.5]; *P* = .02), while those losing pediatric wards (median [IQR], 2013: 70.9 [57.6-83.8]; 2021: 67.0 [56.8-82.0]; *P* = .25) or adult wards that accept pediatric patients (median [IQR], 2013: 71.9 [57.5-87.7]; 2021: 69.5 [58.4-84.5]; *P* = .23) also showed declines, although not significant. No significant differences were observed in hospitals without net changes in inpatient capabilities.

## Discussion

In this national cohort completing both 2013 and 2021 NPRP assessments, we found that 37% experienced a net decrease in pediatric inpatient capabilities, aligning with previously reported trends of declining pediatric inpatient resources over time.^6^ Hospitals that lost PICUs experienced significant declines in pediatric readiness, while those gaining pediatric wards and intensive care units (pediatric and neonatal) showed improvements. Since high pediatric readiness (wPRS ≥ 88) is linked to lower mortality in critically ill and injured children,^5^ future research should evaluate how declining inpatient resources affect patient outcomes. Additionally, examining the financial and policy factors behind pediatric unit closures is essential to maintaining access to high-quality pediatric care. This study has limitations, including reliance on self-reported data from ED leaders, which may not capture collective staff knowledge, and absence of specific dates of inpatient changes, limiting understanding of time to impact on pediatric readiness.

## Notes

Supplement 1.
eMethods.
**eTable. **Questions in National Pediatric Readiness Project (NPRP) Assessment About Pediatric Inpatient Capabilities
eReferences.

Supplement 2.
Data Sharing Statement

## References

[1] Whitfill T, Auerbach M, Scherzer DJ, Shi J, Xiang H, Stanley RM. Emergency care for children in the United States: epidemiology and trends over time. J Emerg Med. 2018;55(3):423-434. doi:10.1016/j.jemermed.2018.04.019 29793812

[2] Gausche-Hill M, Ely M, Schmuhl P, . A national assessment of pediatric readiness of emergency departments. JAMA Pediatr. 2015;169(6):527-534. doi:10.1001/jamapediatrics.2015.138 25867088

[3] Faris GW, Marcin JP, Weinstein E. The current state of the pediatric emergency medicine workforce and innovations to improve pediatric care. Clin Pediatr Emerg Med. 2018;19(3):272-281. doi:10.1016/j.cpem.2018.08.003

[4] Remick KE, Hewes HA, Ely M, . National assessment of pediatric readiness of US emergency departments during the COVID-19 pandemic. JAMA Netw Open. 2023;6(7):e2321707. doi:10.1001/jamanetworkopen.2023.21707 37418265 PMC10329204

[5] Newgard CD, Lin A, Malveau S, ; Pediatric Readiness Study Group. Emergency department pediatric readiness and short- and long-term mortality among children receiving emergency care. JAMA Netw Open. 2023;6(1):e2250941. doi:10.1001/jamanetworkopen.2022.50941 36637819 PMC9857584

[6] Cushing AM, Bucholz EM, Chien AT, Rauch DA, Michelson KA. Availability of pediatric inpatient services in the United States. Pediatrics. 2021;148(1):e2020041723. doi:10.1542/peds.2020-041723 34127553 PMC8642812

