# Supplementary material for: Changes in Pediatric Inpatient Capabilities and Emergency Department Pediatric Readiness
Source: JAMA Netw Open. 2025 May 1;8(5):e258277. doi: 10.1001/jamanetworkopen.2025.8277 (PMC12046425; doi:10.1001/jamanetworkopen.2025.8277)
Supplement: Supplement 1. — eMethods. eTable. Questions in National Pediatric Readiness Project (NPRP) Assessment About Pediatric Inpatient Capabilities eReferences. [file jamanetwopen-e258277-s001.pdf]

Supplemental Online Content

Foster AA, Eisenberg J, Crady R, Hewes HA, Remick KE. Changes in pediatric inpatient capabilities and emergency department pediatric readiness. *JAMA Netw Open*. 2025;8(5):e258277. doi:10.1001/jamanetworkopen.2025.8277

eMethods.

eTable. Questions in National Pediatric Readiness Project (NPRP) Assessment About Pediatric Inpatient Capabilities

eReferences.

This supplemental material has been provided by the authors to give readers additional information about their work.

© 2025 Foster AA et al. *JAMA Network Open*.

eMethods

Study Design and Data Source

We conducted a retrospective serial cross-sectional study of two National Pediatric Readiness Project (NPRP) assessments, administered between 1/2013-8/2013 and 5/2021-8/2021. The assessments consisted of 55 (2013) and 92 (2021) web-based questions that were sent to emergency department (ED) leadership across all US states and territories. The questions evaluated adherence to the national guidelines for emergency care of children.[1–3] The administration of NPRP assessments has been previously described.[4,5]

Inclusion Criteria

Out of 5,017 invitations sent for the 2013 NPRP assessment and 5,150 for the 2021 assessment, the response rates were 82.7% (4,149 responses) and 70.8% (3,647 responses), respectively. We analyzed responses from 2,772 participants that completed both assessments with all inpatient capabilities questions answered.

Outcome Measures

The primary exposure of the study was a net change in pediatric inpatient capabilities between 2013 and 2021. The 2013 and 2021 NPRP assessment included questions regarding the hospital's inpatient capabilities (Supplemental Table 1). Inpatient capabilities were defined as presence of any of the following: pediatric ward, nursery, neonatal Intensive care unit (NICU), pediatric intensive care unit (PICU), adult ward that accepts pediatric patients, and adult intensive care unit (ICU) that accepts pediatric patients. Changes were measured for each participating hospital as the net difference in these inpatient unit capabilities between the two assessment periods. Each unit capability was counted equally. The primary outcome measure was the weighted pediatric readiness score (wPRS), developed by a panel of experts and normalized to a 100-point scale based on the perceived important of each question.[5]

Variable Definitions

Hospital geography was classified by US Census Region (urban, suburban, rural, or remote), and region (Northeast, South, West, Island regions). Hospital configuration was defined based on NPRP assessments (General Hospital, Children's Hospital within a General Hospital, Children's Hospital, Critical Access Hospital, Micro-Hospital, Satellite ED, Freestanding ED, other, unknown).[4,5] Additional hospital and ED characteristics include trauma center designation, Indian or Tribal Hospital designation, ED configuration (general ED, separate pediatric ED, pediatric ED, other), ED pediatric annual volume (low <1,800 patients, medium 1,800-4,999 patients, medium-high 5,000-9,999 patients, and high ≥10,000 patients),[4,5] and presence of a nurse and/or physician pediatric champion within the ED, known as a pediatric emergency care coordinator (PECC). [5,6]

Analysis

Hospital and ED characteristics for the study sample were summarized using frequencies and percentages. Differences in pediatric inpatient capabilities between 2013 and 2021 were analyzed using Wilcoxon signed-rank test to assess changes in wPRS. All analyses were conducted in SAS software version 9.4 (SAS Institute, Carry NC). All tests were 2-sided, and a p-value of < 0.05 was considered statistically significant.

© 2025 Foster AA et al. *JAMA Network Open*.

eReferences

1. American Academy of Pediatrics Committee on Pediatric Emergency Medicine, American College of Emergency Physicians, Emergency Nurses Association PC. Joint policy statement-Guidelines for care of children in the emergency department. *J Emerg Nurs*. 2013;39(2):116-131.

2. American Academy of Pediatrics, Committee on Pediatric Emergency Medicine and American College of Emergency Physicians, and Pediatric Committee. Care of children in the emergency department: guidelines for preparedness. Pediatrics. 2001 Apr;107(4):777-81.

3. Remick K, Gausche-Hill M, Joseph MM, Brown K, Snow SK, Wright JL. Pediatric readiness in the emergency department. *Pediatrics*. 2018;142(5).

4. Remick KE, Hewes HA, Ely M, et al. National assessment of pediatric readiness of US emergency departments during the COVID-19 pandemic. *JAMA Netw Open*. 2023;6(7):e2321707.

5. Gausche-Hill M, Ely M, Schmuhl P, et al. A national assessment of pediatric readiness of emergency departments. *JAMA Pediatr*. 2015;169(6):527-534.

© 2025 Foster AA et al. *JAMA Network Open*.

eTable. Questions in National Pediatric Readiness Project (NPRP) Assessment About Pediatric Inpatient Capabilities

Question	Answer Options	2013 NPRP Assessment	2021 NPRP Assessment
At what trauma level if your hospital currently designated for children?	Pediatric Level I, Pediatric Level II, None of the above	Absent	Present
Which of the following inpatient services does your hospital have on-site…Newborn nursery	Yes, No	Present	Present
Which of the following inpatient services does your hospital have on-site…Neonatal Intensive Care Unit	Yes, No	Present	Present
Which of the following inpatient services does your hospital have on-site…Pediatric Intensive Care Unit	Yes, No	Present	Present
Which of the following inpatient services does your hospital have on-site…Pediatric Step-down unit	Yes, No	Absent	Present
Which of the following inpatient services does your hospital have on-site…Pediatric inpatient ward	Yes, No	Present	Present
Does your hospital ever admit children to the adult intensive care unit (medical or surgical)	Yes, No	Present	Present
Does your hospital ever admit children to the adult step-down unit?	Yes, No	Absent	Present
Does your hospital ever admit children to the adult inpatient ward?	Yes, No	Present	Present

© 2025 Foster AA et al. *JAMA Network Open*.
