# Supplementary material for: Changes in Pediatric Inpatient Capabilities and Emergency Department Pediatric Readiness
Source: JAMA Netw Open. 2025 May 1;8(5):e258277. doi: 10.1001/jamanetworkopen.2025.8277 (PMC12046425; doi:10.1001/jamanetworkopen.2025.8277)
Supplement: Supplement 2. — Data Sharing Statement [file jamanetwopen-e258277-s002.pdf]

Data Sharing Statement

Foster. Changes in Pediatric Inpatient Capabilities and Emergency Department Pediatric Readiness. *JAMA Netw Open*. Published May 01, 2025.
doi:10.1001/jamanetworkopen.2025.8277

Data
Data available: No

Additional Information
Explanation for why data not available: We will not be sharing the data. Data from the National Pediatric Readiness Project cannot be publicly shared to protect responses from participants, who were assured responses would be anonymous and reported in aggregate.
